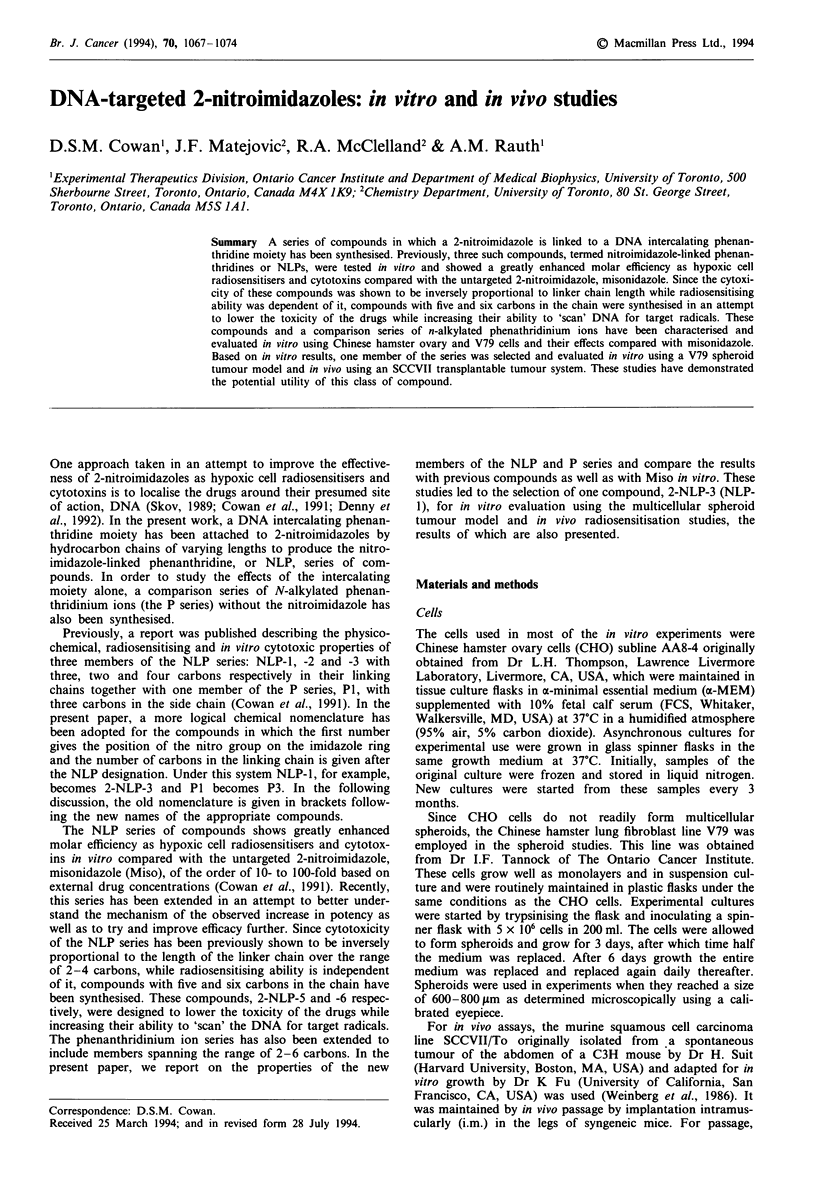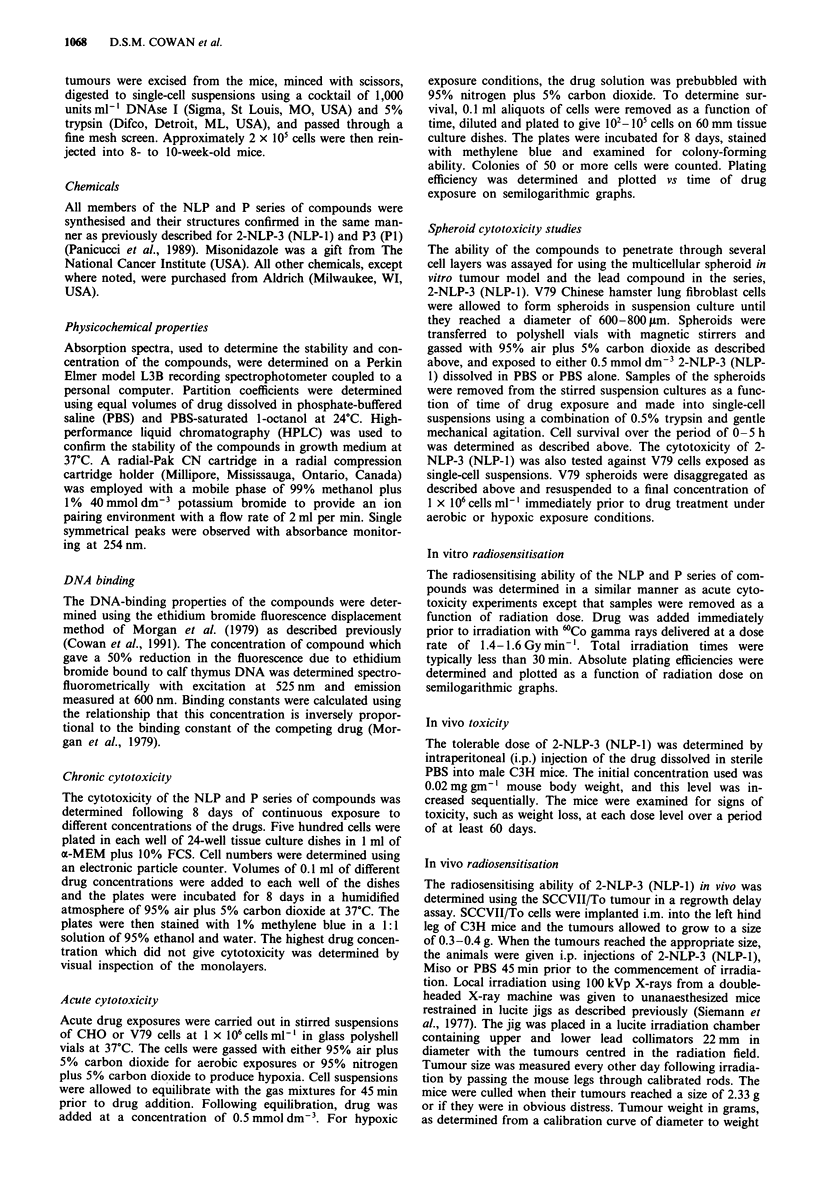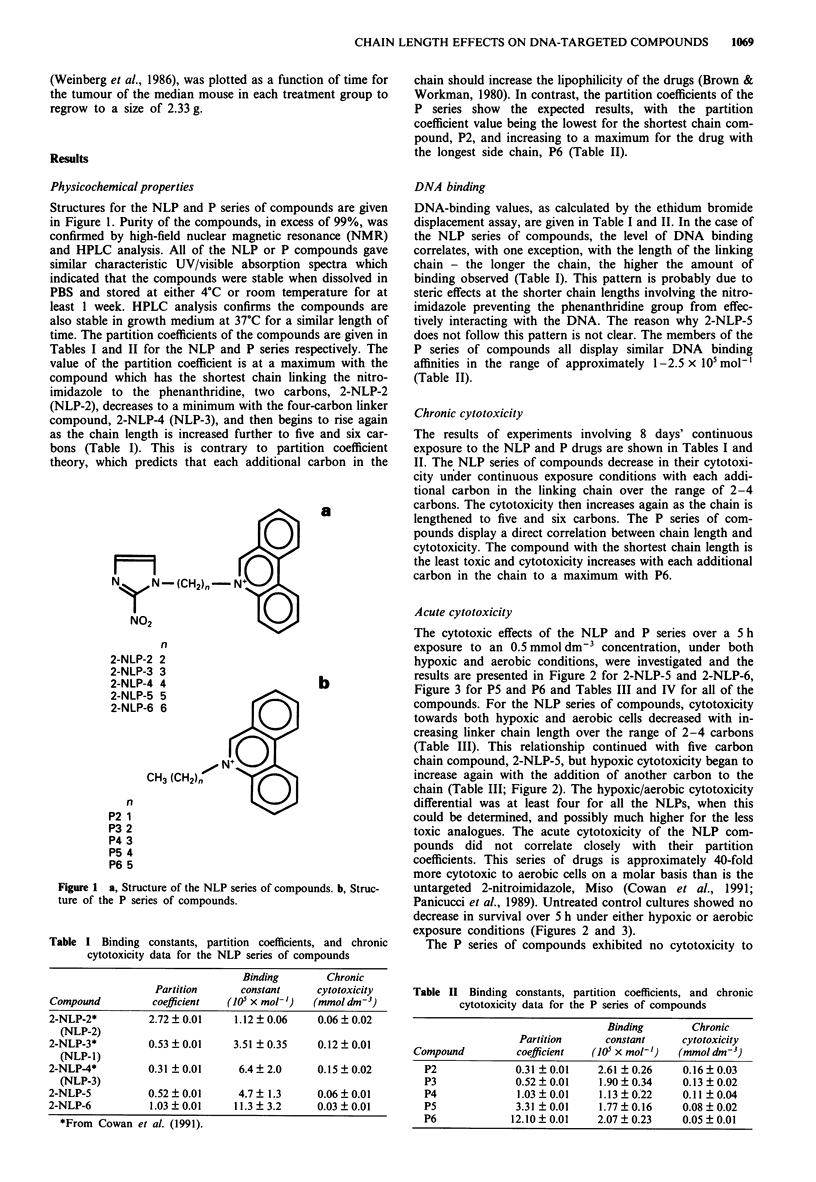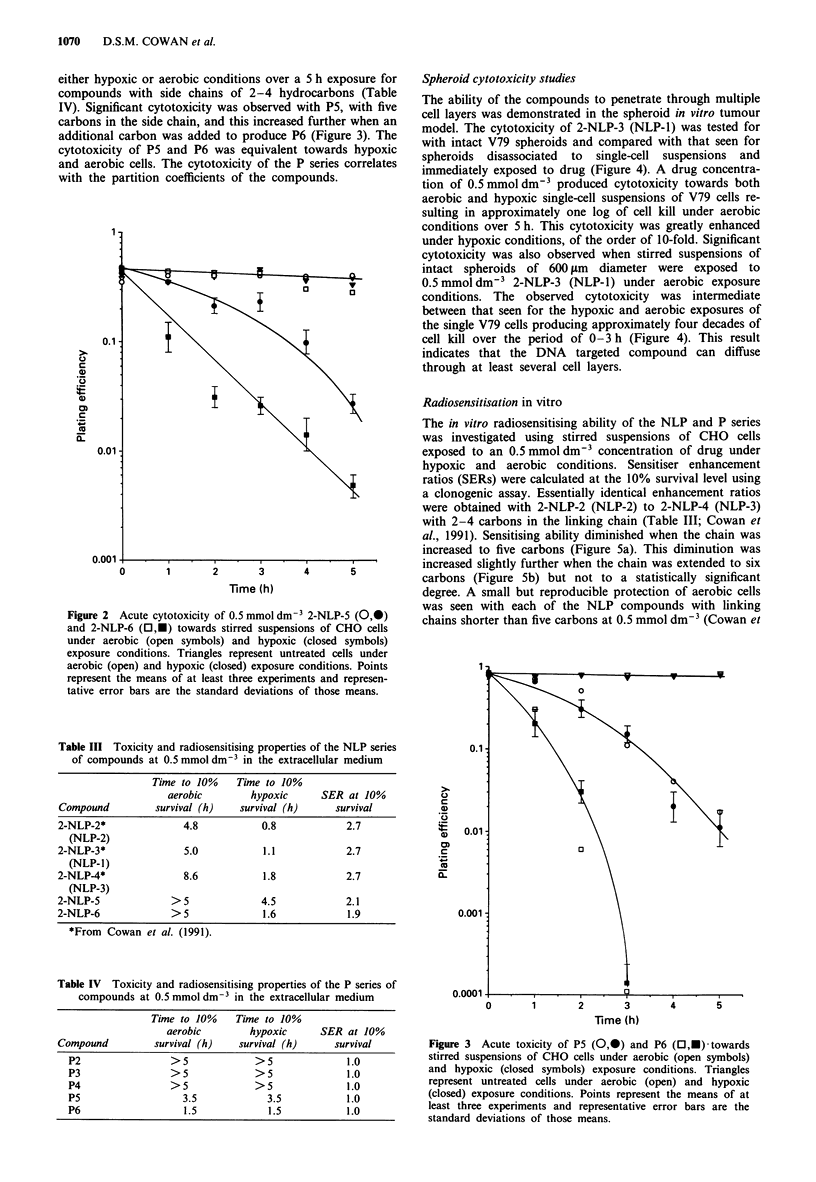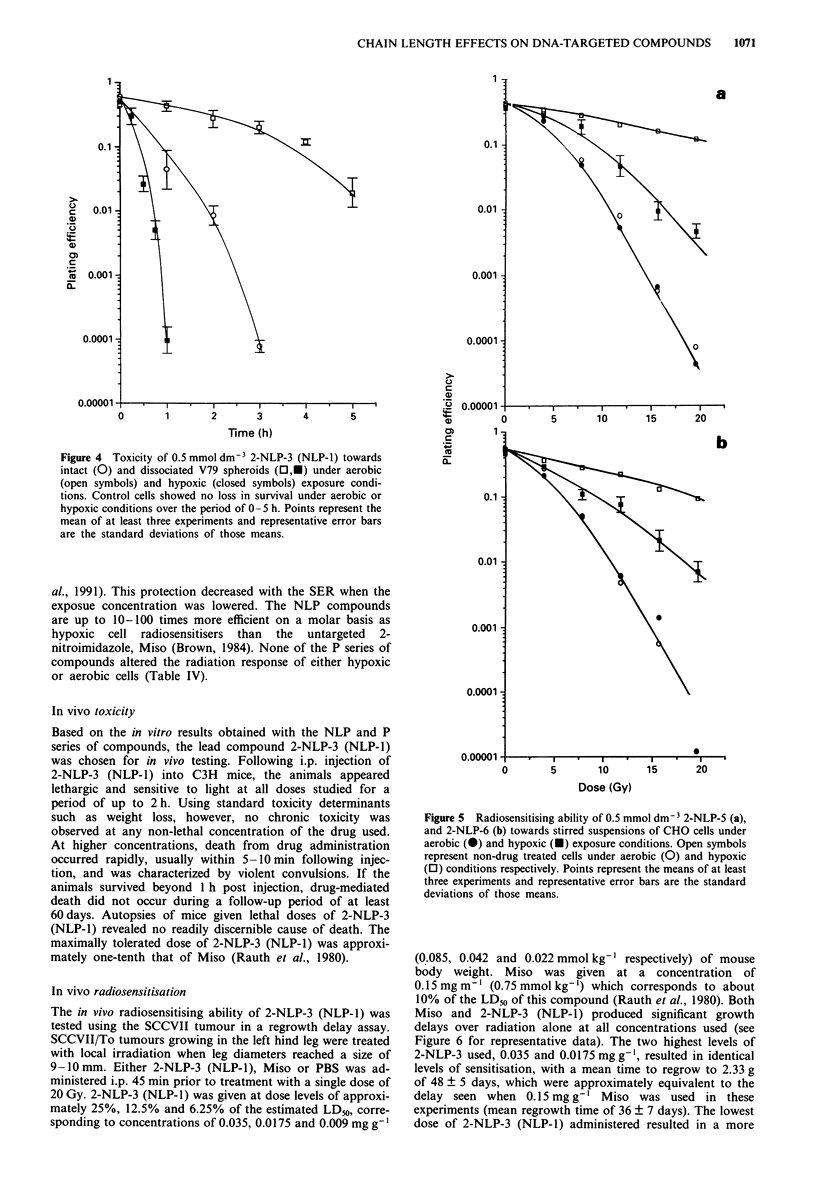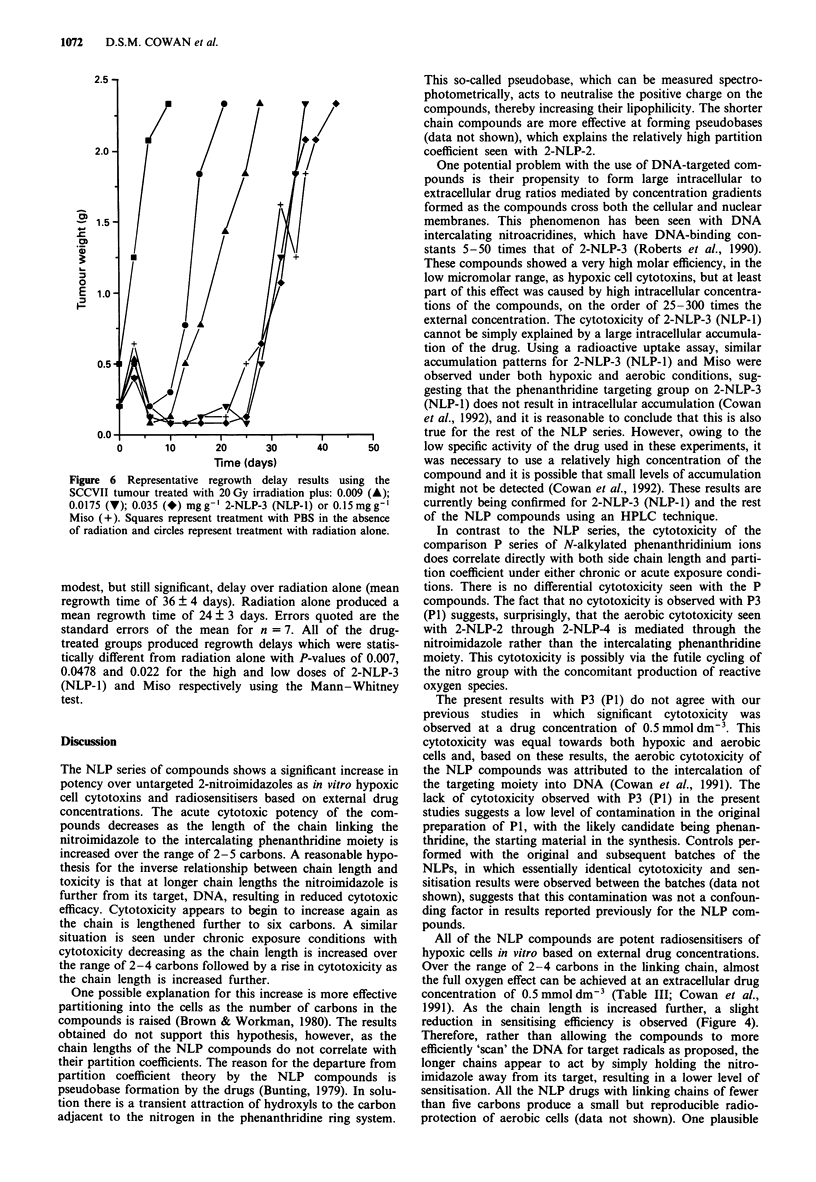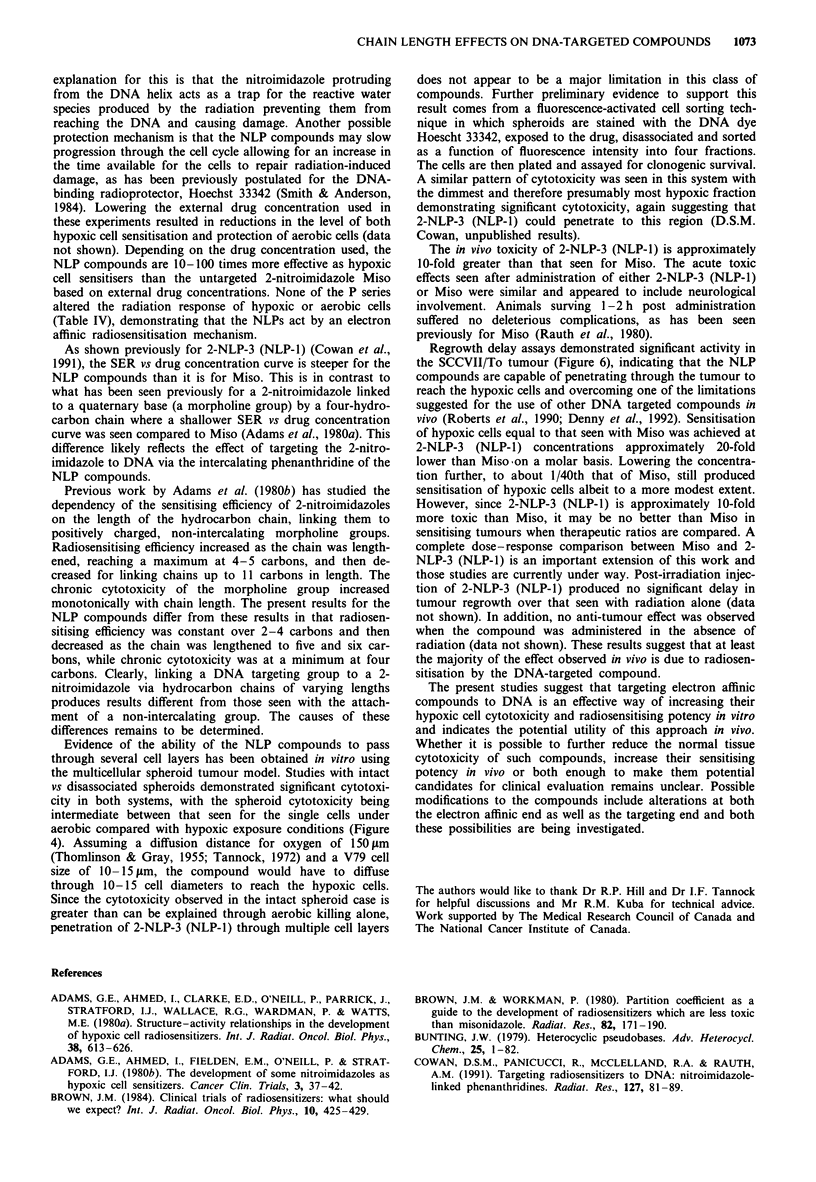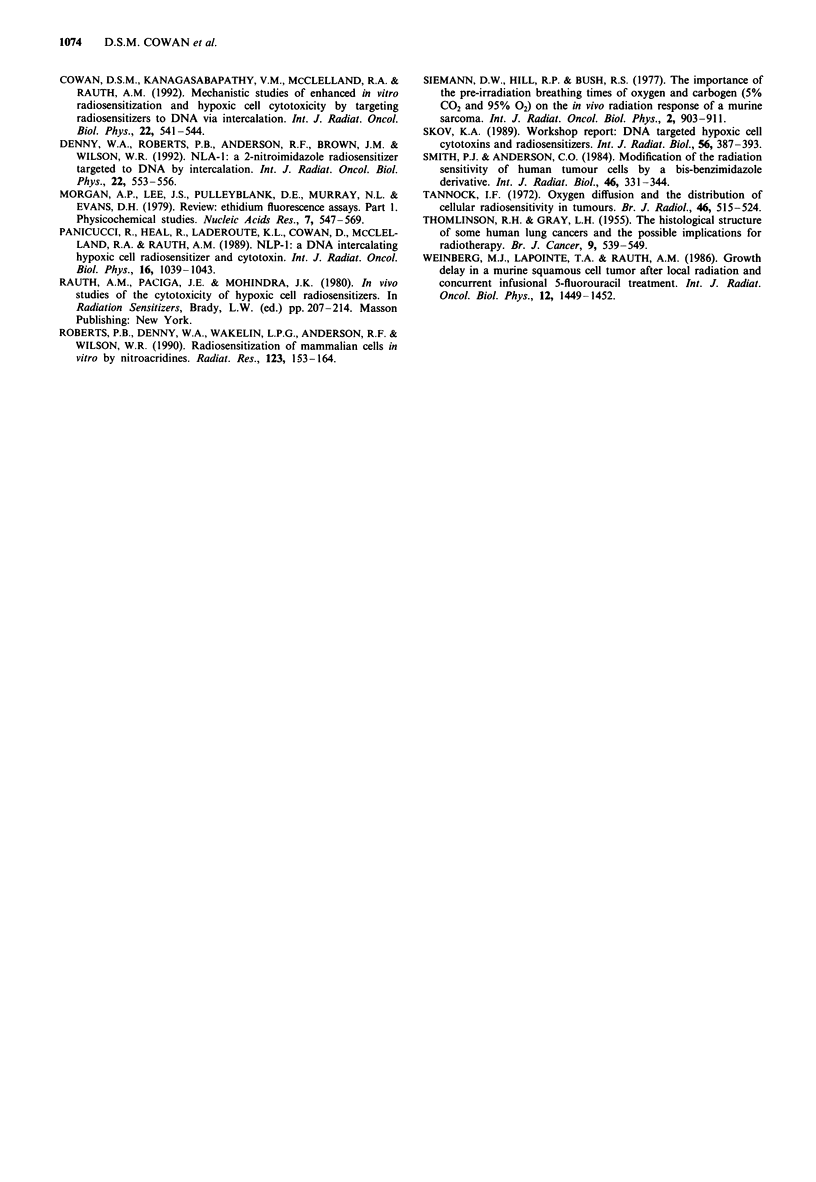# DNA-targeted 2-nitroimidazoles: in vitro and in vivo studies.

**DOI:** 10.1038/bjc.1994.450

**Published:** 1994-12

**Authors:** D. S. Cowan, J. F. Matejovic, R. A. McClelland, A. M. Rauth

**Affiliations:** Experimental Therapeutics Division, Ontario Cancer Institute, Toronto, Canada.

## Abstract

A series of compounds in which a 2-nitroimidazole is linked to a DNA intercalating phenanthridine moiety has been synthesised. Previously, three such compounds, termed nitroimidazole-linked phenanthridines or NLPs, were tested in vitro and showed a greatly enhanced molar efficiency as hypoxic cell radiosensitisers and cytotoxins compared with the untargeted 2-nitroimidazole, misonidazole. Since the cytoxicity of these compounds was shown to be inversely proportional to linker chain length while radiosensitising ability was dependent of it, compounds with five and six carbons in the chain were synthesised in an attempt to lower the toxicity of the drugs while increasing their ability to 'scan' DNA for target radicals. These compounds and a comparison series of n-alkylated phenathridinium ions have been characterised and evaluated in vitro using Chinese hamster ovary and V79 cells and their effects compared with misonidazole. Based on in vitro results, one member of the series was selected and evaluated in vitro using a V79 spheroid tumour model and in vivo using an SCCVII transplantable tumour system. These studies have demonstrated the potential utility of this class of compound.


*Br. J. Cancer* (1994), **70**, 1067–1074 © Macmillan Press Ltd., 1994

### DNA-targeted 2-nitroimidazoles: *in vitro* and *in vivo* studies

D.S.M. Cowan[1], J.F. Matejovic[2], R.A. McClelland[2] & A.M. Rauth[1]

[1]*Experimental Therapeutics Division, Ontario Cancer Institute and Department of Medical Biophysics, University of Toronto, 500 Sherbourne Street, Toronto, Ontario, Canada M4X 1K9;* [2]*Chemistry Department, University of Toronto, 80 St. George Street, Toronto, Ontario, Canada M5S 1A1.*

**Summary** A series of compounds in which a 2-nitroimidazole is linked to a DNA intercalating phenanthridine moiety has been synthesised. Previously, three such compounds, termed nitroimidazole-linked phenanthridines or NLPs, were tested *in vitro* and showed a greatly enhanced molar efficiency as hypoxic cell radiosensitisers and cytotoxins compared with the untargeted 2-nitroimidazole, misonidazole. Since the cytoxicity of these compounds was shown to be inversely proportional to linker chain length while radiosensitising ability was dependent of it, compounds with five and six carbons in the chain were synthesised in an attempt to lower the toxicity of the drugs while increasing their ability to 'scan' DNA for target radicals. These compounds and a comparison series of *n*-alkylated phenathridinium ions have been characterised and evaluated *in vitro* using Chinese hamster ovary and V79 cells and their effects compared with misonidazole. Based on *in vitro* results, one member of the series was selected and evaluated *in vitro* using a V79 spheroid tumour model and *in vivo* using an SCCVII transplantable tumour system. These studies have demonstrated the potential utility of this class of compound.

One approach taken in an attempt to improve the effectiveness of 2-nitroimidazoles as hypoxic cell radiosensitisers and cytotoxins is to localise the drugs around their presumed site of action, DNA (Skov, 1989; Cowan *et al.*, 1991; Denny *et al.*, 1992). In the present work, a DNA intercalating phenanthridine moiety has been attached to 2-nitroimidazoles by hydrocarbon chains of varying lengths to produce the nitroimidazole-linked phenanthridine, or NLP, series of compounds. In order to study the effects of the intercalating moiety alone, a comparison series of *N*-alkylated phenanthridinium ions (the P series) without the nitroimidazole has also been synthesised.

Previously, a report was published describing the physicochemical, radiosensitising and *in vitro* cytotoxic properties of three members of the NLP series: NLP-1, -2 and -3 with three, two and four carbons respectively in their linking chains together with one member of the P series, P1, with three carbons in the side chain (Cowan *et al.*, 1991). In the present paper, a more logical chemical nomenclature has been adopted for the compounds in which the first number gives the position of the nitro group on the imidazole ring and the number of carbons in the linking chain is given after the NLP designation. Under this system NLP-1, for example, becomes 2-NLP-3 and P1 becomes P3. In the following discussion, the old nomenclature is given in brackets following the new names of the appropriate compounds.

The NLP series of compounds shows greatly enhanced molar efficiency as hypoxic cell radiosensitisers and cytotoxins *in vitro* compared with the untargeted 2-nitroimidazole, misonidazole (Miso), of the order of 10- to 100-fold based on external drug concentrations (Cowan *et al.*, 1991). Recently, this series has been extended in an attempt to better understand the mechanism of the observed increase in potency as well as to try and improve efficacy further. Since cytotoxicity of the NLP series has been previously shown to be inversely proportional to the length of the linker chain over the range of 2–4 carbons, while radiosensitising ability is independent of it, compounds with five and six carbons in the chain have been synthesised. These compounds, 2-NLP-5 and -6 respectively, were designed to lower the toxicity of the drugs while increasing their ability to 'scan' the DNA for target radicals. The phenanthridinium ion series has also been extended to include members spanning the range of 2–6 carbons. In the present paper, we report on the properties of the new members of the NLP and P series and compare the results with previous compounds as well as with Miso *in vitro*. These studies led to the selection of one compound, 2-NLP-3 (NLP-1), for *in vitro* evaluation using the multicellular spheroid tumour model and *in vivo* radiosensitisation studies, the results of which are also presented.

### Materials and methods

### *Cells*

The cells used in most of the *in vitro* experiments were Chinese hamster ovary cells (CHO) subline AA8-4 originally obtained from Dr L.H. Thompson, Lawrence Livermore Laboratory, Livermore, CA, USA, which were maintained in tissue culture flasks in α-minimal essential medium (α-MEM) supplemented with 10% fetal calf serum (FCS, Whitaker, Walkersville, MD, USA) at 37°C in a humidified atmosphere (95% air, 5% carbon dioxide). Asynchronous cultures for experimental use were grown in glass spinner flasks in the same growth medium at 37°C. Initially, samples of the original culture were frozen and stored in liquid nitrogen. New cultures were started from these samples every 3 months.

Since CHO cells do not readily form multicellular spheroids, the Chinese hamster lung fibroblast line V79 was employed in the spheroid studies. This line was obtained from Dr I.F. Tannock of The Ontario Cancer Institute. These cells grow well as monolayers and in suspension culture and were routinely maintained in plastic flasks under the same conditions as the CHO cells. Experimental cultures were started by trypsinising the flask and inoculating a spinner flask with $5 \times 10^6$ cells in 200 ml. The cells were allowed to form spheroids and grow for 3 days, after which time half the medium was replaced. After 6 days growth the entire medium was replaced and replaced again daily thereafter. Spheroids were used in experiments when they reached a size of 600–800 μm as determined microscopically using a calibrated eyepiece.

For *in vivo* assays, the murine squamous cell carcinoma line SCCVII/To originally isolated from a spontaneous tumour of the abdomen of a C3H mouse by Dr H. Suit (Harvard University, Boston, MA, USA) and adapted for *in vitro* growth by Dr K Fu (University of California, San Francisco, CA, USA) was used (Weinberg *et al.*, 1986). It was maintained by *in vivo* passage by implantation intramuscularly (i.m.) in the legs of syngeneic mice. For passage,

Correspondence: D.S.M. Cowan.
Received 25 March 1994; and in revised form 28 July 1994.

tumours were excised from the mice, minced with scissors, digested to single-cell suspensions using a cocktail of 1,000 units $ml^{-1}$ DNAse I (Sigma, St Louis, MO, USA) and 5% trypsin (Difco, Detroit, ML, USA), and passed through a fine mesh screen. Approximately $2 \times 10^5$ cells were then reinjected into 8- to 10-week-old mice.

*Chemicals*

All members of the NLP and P series of compounds were synthesised and their structures confirmed in the same manner as previously described for 2-NLP-3 (NLP-1) and P3 (P1) (Panicucci *et al.*, 1989). Misonidazole was a gift from The National Cancer Institute (USA). All other chemicals, except where noted, were purchased from Aldrich (Milwaukee, WI, USA).

*Physicochemical properties*

Absorption spectra, used to determine the stability and concentration of the compounds, were determined on a Perkin Elmer model L3B recording spectrophotometer coupled to a personal computer. Partition coefficients were determined using equal volumes of drug dissolved in phosphate-buffered saline (PBS) and PBS-saturated 1-octanol at 24°C. High-performance liquid chromatography (HPLC) was used to confirm the stability of the compounds in growth medium at 37°C. A radial-Pak CN cartridge in a radial compression cartridge holder (Millipore, Mississauga, Ontario, Canada) was employed with a mobile phase of 99% methanol plus 1% 40 mmol $dm^{-3}$ potassium bromide to provide an ion pairing environment with a flow rate of 2 ml per min. Single symmetrical peaks were observed with absorbance monitoring at 254 nm.

*DNA binding*

The DNA-binding properties of the compounds were determined using the ethidium bromide fluorescence displacement method of Morgan *et al.* (1979) as described previously (Cowan *et al.*, 1991). The concentration of compound which gave a 50% reduction in the fluorescence due to ethidium bromide bound to calf thymus DNA was determined spectrofluorometrically with excitation at 525 nm and emission measured at 600 nm. Binding constants were calculated using the relationship that this concentration is inversely proportional to the binding constant of the competing drug (Morgan *et al.*, 1979).

*Chronic cytotoxicity*

The cytotoxicity of the NLP and P series of compounds was determined following 8 days of continuous exposure to different concentrations of the drugs. Five hundred cells were plated in each well of 24-well tissue culture dishes in 1 ml of α-MEM plus 10% FCS. Cell numbers were determined using an electronic particle counter. Volumes of 0.1 ml of different drug concentrations were added to each well of the dishes and the plates were incubated for 8 days in a humidified atmosphere of 95% air plus 5% carbon dioxide at 37°C. The plates were then stained with 1% methylene blue in a 1:1 solution of 95% ethanol and water. The highest drug concentration which did not give cytotoxicity was determined by visual inspection of the monolayers.

*Acute cytotoxicity*

Acute drug exposures were carried out in stirred suspensions of CHO or V79 cells at $1 \times 10^6$ cells $ml^{-1}$ in glass polyshell vials at 37°C. The cells were gassed with either 95% air plus 5% carbon dioxide for aerobic exposures or 95% nitrogen plus 5% carbon dioxide to produce hypoxia. Cell suspensions were allowed to equilibrate with the gas mixtures for 45 min prior to drug addition. Following equilibration, drug was added at a concentration of 0.5 mmol $dm^{-3}$. For hypoxic exposure conditions, the drug solution was prebubbled with 95% nitrogen plus 5% carbon dioxide. To determine survival, 0.1 ml aliquots of cells were removed as a function of time, diluted and plated to give $10^2-10^5$ cells on 60 mm tissue culture dishes. The plates were incubated for 8 days, stained with methylene blue and examined for colony-forming ability. Colonies of 50 or more cells were counted. Plating efficiency was determined and plotted *vs* time of drug exposure on semilogarithmic graphs.

*Spheroid cytotoxicity studies*

The ability of the compounds to penetrate through several cell layers was assayed for using the multicellular spheroid *in vitro* tumour model and the lead compound in the series, 2-NLP-3 (NLP-1). V79 Chinese hamster lung fibroblast cells were allowed to form spheroids in suspension culture until they reached a diameter of 600–800 μm. Spheroids were transferred to polyshell vials with magnetic stirrers and gassed with 95% air plus 5% carbon dioxide as described above, and exposed to either 0.5 mmol $dm^{-3}$ 2-NLP-3 (NLP-1) dissolved in PBS or PBS alone. Samples of the spheroids were removed from the stirred suspension cultures as a function of time of drug exposure and made into single-cell suspensions using a combination of 0.5% trypsin and gentle mechanical agitation. Cell survival over the period of 0–5 h was determined as described above. The cytotoxicity of 2-NLP-3 (NLP-1) was also tested against V79 cells exposed as single-cell suspensions. V79 spheroids were disaggregated as described above and resuspended to a final concentration of $1 \times 10^6$ cells $ml^{-1}$ immediately prior to drug treatment under aerobic or hypoxic exposure conditions.

In vitro *radiosensitisation*

The radiosensitising ability of the NLP and P series of compounds was determined in a similar manner as acute cytotoxicity experiments except that samples were removed as a function of radiation dose. Drug was added immediately prior to irradiation with $^{60}Co$ gamma rays delivered at a dose rate of 1.4–1.6 Gy $min^{-1}$. Total irradiation times were typically less than 30 min. Absolute plating efficiencies were determined and plotted as a function of radiation dose on semilogarithmic graphs.

In vivo *toxicity*

The tolerable dose of 2-NLP-3 (NLP-1) was determined by intraperitoneal (i.p.) injection of the drug dissolved in sterile PBS into male C3H mice. The initial concentration used was 0.02 mg $gm^{-1}$ mouse body weight, and this level was increased sequentially. The mice were examined for signs of toxicity, such as weight loss, at each dose level over a period of at least 60 days.

In vivo *radiosensitisation*

The radiosensitising ability of 2-NLP-3 (NLP-1) *in vivo* was determined using the SCCVII/To tumour in a regrowth delay assay. SCCVII/To cells were implanted i.m. into the left hind leg of C3H mice and the tumours allowed to grow to a size of 0.3–0.4 g. When the tumours reached the appropriate size, the animals were given i.p. injections of 2-NLP-3 (NLP-1), Miso or PBS 45 min prior to the commencement of irradiation. Local irradiation using 100 kVp X-rays from a double-headed X-ray machine was given to unanaesthesized mice restrained in lucite jigs as described previously (Siemann *et al.*, 1977). The jig was placed in a lucite irradiation chamber containing upper and lower lead collimators 22 mm in diameter with the tumours centred in the radiation field. Tumour size was measured every other day following irradiation by passing the mouse legs through calibrated rods. The mice were culled when their tumours reached a size of 2.33 g or if they were in obvious distress. Tumour weight in grams, as determined from a calibration curve of diameter to weight

(Weinberg *et al.*, 1986), was plotted as a function of time for the tumour of the median mouse in each treatment group to regrow to a size of 2.33 g.

### Results

### *Physicochemical properties*

Structures for the NLP and P series of compounds are given in Figure 1. Purity of the compounds, in excess of 99%, was confirmed by high-field nuclear magnetic resonance (NMR) and HPLC analysis. All of the NLP or P compounds gave similar characteristic UV/visible absorption spectra which indicated that the compounds were stable when dissolved in PBS and stored at either 4°C or room temperature for at least 1 week. HPLC analysis confirms the compounds are also stable in growth medium at 37°C for a similar length of time. The partition coefficients of the compounds are given in Tables I and II for the NLP and P series respectively. The value of the partition coefficient is at a maximum with the compound which has the shortest chain linking the nitroimidazole to the phenanthridine, two carbons, 2-NLP-2 (NLP-2), decreases to a minimum with the four-carbon linker compound, 2-NLP-4 (NLP-3), and then begins to rise again as the chain length is increased further to five and six carbons (Table I). This is contrary to partition coefficient theory, which predicts that each additional carbon in the chain should increase the lipophilicity of the drugs (Brown & Workman, 1980). In contrast, the partition coefficients of the P series show the expected results, with the partition coefficient value being the lowest for the shortest chain compound, P2, and increasing to a maximum for the drug with the longest side chain, P6 (Table II).

**Figure 1** **a**, Structure of the NLP series of compounds. **b**, Structure of the P series of compounds.

**Table I** Binding constants, partition coefficients, and chronic cytotoxicity data for the NLP series of compounds

| *Compound* | *Partition coefficient* | *Binding constant ($10^5 \times mol^{-1}$)* | *Chronic cytotoxicity (mmol dm$^{-3}$)* |
|---|---|---|---|
| 2-NLP-2* (NLP-2) | 2.72 ± 0.01 | 1.12 ± 0.06 | 0.06 ± 0.02 |
| 2-NLP-3* (NLP-1) | 0.53 ± 0.01 | 3.51 ± 0.35 | 0.12 ± 0.01 |
| 2-NLP-4* (NLP-3) | 0.31 ± 0.01 | 6.4 ± 2.0 | 0.15 ± 0.02 |
| 2-NLP-5 | 0.52 ± 0.01 | 4.7 ± 1.3 | 0.06 ± 0.01 |
| 2-NLP-6 | 1.03 ± 0.01 | 11.3 ± 3.2 | 0.03 ± 0.01 |

*From Cowan *et al.* (1991).

### *DNA binding*

DNA-binding values, as calculated by the ethidum bromide displacement assay, are given in Table I and II. In the case of the NLP series of compounds, the level of DNA binding correlates, with one exception, with the length of the linking chain – the longer the chain, the higher the amount of binding observed (Table I). This pattern is probably due to steric effects at the shorter chain lengths involving the nitroimidazole preventing the phenanthridine group from effectively interacting with the DNA. The reason why 2-NLP-5 does not follow this pattern is not clear. The members of the P series of compounds all display similar DNA binding affinities in the range of approximately $1-2.5 \times 10^5 mol^{-1}$ (Table II).

### *Chronic cytotoxicity*

The results of experiments involving 8 days' continuous exposure to the NLP and P drugs are shown in Tables I and II. The NLP series of compounds decrease in their cytotoxicity under continuous exposure conditions with each additional carbon in the linking chain over the range of 2–4 carbons. The cytotoxicity then increases again as the chain is lengthened to five and six carbons. The P series of compounds display a direct correlation between chain length and cytotoxicity. The compound with the shortest chain length is the least toxic and cytotoxicity increases with each additional carbon in the chain to a maximum with P6.

### *Acute cytotoxicity*

The cytotoxic effects of the NLP and P series over a 5 h exposure to an 0.5 mmol dm$^{-3}$ concentration, under both hypoxic and aerobic conditions, were investigated and the results are presented in Figure 2 for 2-NLP-5 and 2-NLP-6, Figure 3 for P5 and P6 and Tables III and IV for all of the compounds. For the NLP series of compounds, cytotoxicity towards both hypoxic and aerobic cells decreased with increasing linker chain length over the range of 2–4 carbons (Table III). This relationship continued with five carbon chain compound, 2-NLP-5, but hypoxic cytotoxicity began to increase again with the addition of another carbon to the chain (Table III; Figure 2). The hypoxic/aerobic cytotoxicity differential was at least four for all the NLPs, when this could be determined, and possibly much higher for the less toxic analogues. The acute cytotoxicity of the NLP compounds did not correlate closely with their partition coefficients. This series of drugs is approximately 40-fold more cytotoxic to aerobic cells on a molar basis than is the untargeted 2-nitroimidazole, Miso (Cowan *et al.*, 1991; Panicucci *et al.*, 1989). Untreated control cultures showed no decrease in survival over 5 h under either hypoxic or aerobic exposure conditions (Figures 2 and 3).

The P series of compounds exhibited no cytotoxicity to

**Table II** Binding constants, partition coefficients, and chronic cytotoxicity data for the P series of compounds

| *Compound* | *Partition coefficient* | *Binding constant ($10^5 \times mol^{-1}$)* | *Chronic cytotoxicity (mmol dm$^{-3}$)* |
|---|---|---|---|
| P2 | 0.31 ± 0.01 | 2.61 ± 0.26 | 0.16 ± 0.03 |
| P3 | 0.52 ± 0.01 | 1.90 ± 0.34 | 0.13 ± 0.02 |
| P4 | 1.03 ± 0.01 | 1.13 ± 0.22 | 0.11 ± 0.04 |
| P5 | 3.31 ± 0.01 | 1.77 ± 0.16 | 0.08 ± 0.02 |
| P6 | 12.10 ± 0.01 | 2.07 ± 0.23 | 0.05 ± 0.01 |

either hypoxic or aerobic conditions over a 5 h exposure for compounds with side chains of 2–4 hydrocarbons (Table IV). Significant cytotoxicity was observed with P5, with five carbons in the side chain, and this increased further when an additional carbon was added to produce P6 (Figure 3). The cytotoxicity of P5 and P6 was equivalent towards hypoxic and aerobic cells. The cytotoxicity of the P series correlates with the partition coefficients of the compounds.

**Figure 2** Acute cytotoxicity of 0.5 mmol dm$^{-3}$ 2-NLP-5 (O,●) and 2-NLP-6 (□,■) towards stirred suspensions of CHO cells under aerobic (open symbols) and hypoxic (closed symbols) exposure conditions. Triangles represent untreated cells under aerobic (open) and hypoxic (closed) exposure conditions. Points represent the means of at least three experiments and representative error bars are the standard deviations of those means.

**Table III** Toxicity and radiosensitising properties of the NLP series of compounds at 0.5 mmol dm$^{-3}$ in the extracellular medium

| Compound | *Time to 10% aerobic survival (h)* | *Time to 10% hypoxic survival (h)* | *SER at 10% survival* |
|---|---|---|---|
| 2-NLP-2* (NLP-2) | 4.8 | 0.8 | 2.7 |
| 2-NLP-3* (NLP-1) | 5.0 | 1.1 | 2.7 |
| 2-NLP-4* (NLP-3) | 8.6 | 1.8 | 2.7 |
| 2-NLP-5 | >5 | 4.5 | 2.1 |
| 2-NLP-6 | >5 | 1.6 | 1.9 |

*From Cowan *et al.* (1991).

**Table IV** Toxicity and radiosensitising properties of the P series of compounds at 0.5 mmol dm$^{-3}$ in the extracellular medium

| Compound | *Time to 10% aerobic survival (h)* | *Time to 10% hypoxic survival (h)* | *SER at 10% survival* |
|---|---|---|---|
| P2 | >5 | >5 | 1.0 |
| P3 | >5 | >5 | 1.0 |
| P4 | >5 | >5 | 1.0 |
| P5 | 3.5 | 3.5 | 1.0 |
| P6 | 1.5 | 1.5 | 1.0 |

*Spheroid cytotoxicity studies*

The ability of the compounds to penetrate through multiple cell layers was demonstrated in the spheroid *in vitro* tumour model. The cytotoxicity of 2-NLP-3 (NLP-1) was tested for with intact V79 spheroids and compared with that seen for spheroids disassociated to single-cell suspensions and immediately exposed to drug (Figure 4). A drug concentration of 0.5 mmol dm$^{-3}$ produced cytotoxicity towards both aerobic and hypoxic single-cell suspensions of V79 cells resulting in approximately one log of cell kill under aerobic conditions over 5 h. This cytotoxicity was greatly enhanced under hypoxic conditions, of the order of 10-fold. Significant cytotoxicity was also observed when stirred suspensions of intact spheroids of 600 µm diameter were exposed to 0.5 mmol dm$^{-3}$ 2-NLP-3 (NLP-1) under aerobic exposure conditions. The observed cytotoxicity was intermediate between that seen for the hypoxic and aerobic exposures of the single V79 cells producing approximately four decades of cell kill over the period of 0–3 h (Figure 4). This result indicates that the DNA targeted compound can diffuse through at least several cell layers.

*Radiosensitisation* in vitro

The *in vitro* radiosensitising ability of the NLP and P series was investigated using stirred suspensions of CHO cells exposed to an 0.5 mmol dm$^{-3}$ concentration of drug under hypoxic and aerobic conditions. Sensitiser enhancement ratios (SERs) were calculated at the 10% survival level using a clonogenic assay. Essentially identical enhancement ratios were obtained with 2-NLP-2 (NLP-2) to 2-NLP-4 (NLP-3) with 2–4 carbons in the linking chain (Table III; Cowan *et al.*, 1991). Sensitising ability diminished when the chain was increased to five carbons (Figure 5a). This diminution was increased slightly further when the chain was extended to six carbons (Figure 5b) but not to a statistically significant degree. A small but reproducible protection of aerobic cells was seen with each of the NLP compounds with linking chains shorter than five carbons at 0.5 mmol dm$^{-3}$ (Cowan *et*

**Figure 3** Acute toxicity of P5 (O,●) and P6 (□,■) towards stirred suspensions of CHO cells under aerobic (open symbols) and hypoxic (closed symbols) exposure conditions. Triangles represent untreated cells under aerobic (open) and hypoxic (closed) exposure conditions. Points represent the means of at least three experiments and representative error bars are the standard deviations of those means.

**Figure 4** Toxicity of 0.5 mmol dm$^{-3}$ 2-NLP-3 (NLP-1) towards intact (○) and dissociated V79 spheroids (□,■) under aerobic (open symbols) and hypoxic (closed symbols) exposure conditions. Control cells showed no loss in survival under aerobic or hypoxic conditions over the period of 0–5 h. Points represent the mean of at least three experiments and representative error bars are the standard deviations of those means.

*al.*, 1991). This protection decreased with the SER when the exposue concentration was lowered. The NLP compounds are up to 10–100 times more efficient on a molar basis as hypoxic cell radiosensitisers than the untargeted 2-nitroimidazole, Miso (Brown, 1984). None of the P series of compounds altered the radiation response of either hypoxic or aerobic cells (Table IV).

### In vivo *toxicity*

Based on the *in vitro* results obtained with the NLP and P series of compounds, the lead compound 2-NLP-3 (NLP-1) was chosen for *in vivo* testing. Following i.p. injection of 2-NLP-3 (NLP-1) into C3H mice, the animals appeared lethargic and sensitive to light at all doses studied for a period of up to 2 h. Using standard toxicity determinants such as weight loss, however, no chronic toxicity was observed at any non-lethal concentration of the drug used. At higher concentrations, death from drug administration occurred rapidly, usually within 5–10 min following injection, and was characterized by violent convulsions. If the animals survived beyond 1 h post injection, drug-mediated death did not occur during a follow-up period of at least 60 days. Autopsies of mice given lethal doses of 2-NLP-3 (NLP-1) revealed no readily discernible cause of death. The maximally tolerated dose of 2-NLP-3 (NLP-1) was approximately one-tenth that of Miso (Rauth *et al.*, 1980).

### In vivo *radiosensitisation*

The *in vivo* radiosensitising ability of 2-NLP-3 (NLP-1) was tested using the SCCVII tumour in a regrowth delay assay. SCCVII/To tumours growing in the left hind leg were treated with local irradiation when leg diameters reached a size of 9–10 mm. Either 2-NLP-3 (NLP-1), Miso or PBS was administered i.p. 45 min prior to treatment with a single dose of 20 Gy. 2-NLP-3 (NLP-1) was given at dose levels of approximately 25%, 12.5% and 6.25% of the estimated $LD_{50}$, corresponding to concentrations of 0.035, 0.0175 and 0.009 mg g$^{-1}$

**Figure 5** Radiosensitising ability of 0.5 mmol dm$^{-3}$ 2-NLP-5 (**a**), and 2-NLP-6 (**b**) towards stirred suspensions of CHO cells under aerobic (●) and hypoxic (■) exposure conditions. Open symbols represent non-drug treated cells under aerobic (○) and hypoxic (□) conditions respectively. Points represent the means of at least three experiments and representative error bars are the standard deviations of those means.

(0.085, 0.042 and 0.022 mmol kg$^{-1}$ respectively) of mouse body weight. Miso was given at a concentration of 0.15 mg m$^{-1}$ (0.75 mmol kg$^{-1}$) which corresponds to about 10% of the $LD_{50}$ of this compound (Rauth *et al.*, 1980). Both Miso and 2-NLP-3 (NLP-1) produced significant growth delays over radiation alone at all concentrations used (see Figure 6 for representative data). The two highest levels of 2-NLP-3 used, 0.035 and 0.0175 mg g$^{-1}$, resulted in identical levels of sensitisation, with a mean time to regrow to 2.33 g of 48 ± 5 days, which were approximately equivalent to the delay seen when 0.15 mg g$^{-1}$ Miso was used in these experiments (mean regrowth time of 36 ± 7 days). The lowest dose of 2-NLP-3 (NLP-1) administered resulted in a more

**Figure 6** Representative regrowth delay results using the SCCVII tumour treated with 20 Gy irradiation plus: 0.009 (▲); 0.0175 (▼); 0.035 (◆) mg $g^{-1}$ 2-NLP-3 (NLP-1) or 0.15 mg $g^{-1}$ Miso (+). Squares represent treatment with PBS in the absence of radiation and circles represent treatment with radiation alone.

modest, but still significant, delay over radiation alone (mean regrowth time of 36 ± 4 days). Radiation alone produced a mean regrowth time of 24 ± 3 days. Errors quoted are the standard errors of the mean for $n = 7$. All of the drug-treated groups produced regrowth delays which were statistically different from radiation alone with $P$-values of 0.007, 0.0478 and 0.022 for the high and low doses of 2-NLP-3 (NLP-1) and Miso respectively using the Mann–Whitney test.

### Discussion

The NLP series of compounds shows a significant increase in potency over untargeted 2-nitroimidazoles as *in vitro* hypoxic cell cytotoxins and radiosensitisers based on external drug concentrations. The acute cytotoxic potency of the compounds decreases as the length of the chain linking the nitroimidazole to the intercalating phenanthridine moiety is increased over the range of 2–5 carbons. A reasonable hypothesis for the inverse relationship between chain length and toxicity is that at longer chain lengths the nitroimidazole is further from its target, DNA, resulting in reduced cytotoxic efficacy. Cytotoxicity appears to begin to increase again as the chain is lengthened further to six carbons. A similar situation is seen under chronic exposure conditions with cytotoxicity decreasing as the chain length is increased over the range of 2–4 carbons followed by a rise in cytotoxicity as the chain length is increased further.

One possible explanation for this increase is more effective partitioning into the cells as the number of carbons in the compounds is raised (Brown & Workman, 1980). The results obtained do not support this hypothesis, however, as the chain lengths of the NLP compounds do not correlate with their partition coefficients. The reason for the departure from partition coefficient theory by the NLP compounds is pseudobase formation by the drugs (Bunting, 1979). In solution there is a transient attraction of hydroxyls to the carbon adjacent to the nitrogen in the phenanthridine ring system. This so-called pseudobase, which can be measured spectrophotometrically, acts to neutralise the positive charge on the compounds, thereby increasing their lipophilicity. The shorter chain compounds are more effective at forming pseudobases (data not shown), which explains the relatively high partition coefficient seen with 2-NLP-2.

One potential problem with the use of DNA-targeted compounds is their propensity to form large intracellular to extracellular drug ratios mediated by concentration gradients formed as the compounds cross both the cellular and nuclear membranes. This phenomenon has been seen with DNA intercalating nitroacridines, which have DNA-binding constants 5–50 times that of 2-NLP-3 (Roberts *et al.*, 1990). These compounds showed a very high molar efficiency, in the low micromolar range, as hypoxic cell cytotoxins, but at least part of this effect was caused by high intracellular concentrations of the compounds, on the order of 25–300 times the external concentration. The cytotoxicity of 2-NLP-3 (NLP-1) cannot be simply explained by a large intracellular accumulation of the drug. Using a radioactive uptake assay, similar accumulation patterns for 2-NLP-3 (NLP-1) and Miso were observed under both hypoxic and aerobic conditions, suggesting that the phenanthridine targeting group on 2-NLP-3 (NLP-1) does not result in intracellular accumulation (Cowan *et al.*, 1992), and it is reasonable to conclude that this is also true for the rest of the NLP series. However, owing to the low specific activity of the drug used in these experiments, it was necessary to use a relatively high concentration of the compound and it is possible that small levels of accumulation might not be detected (Cowan *et al.*, 1992). These results are currently being confirmed for 2-NLP-3 (NLP-1) and the rest of the NLP compounds using an HPLC technique.

In contrast to the NLP series, the cytotoxicity of the comparison P series of *N*-alkylated phenanthridinium ions does correlate directly with both side chain length and partition coefficient under either chronic or acute exposure conditions. There is no differential cytotoxicity seen with the P compounds. The fact that no cytotoxicity is observed with P3 (P1) suggests, surprisingly, that the aerobic cytotoxicity seen with 2-NLP-2 through 2-NLP-4 is mediated through the nitroimidazole rather than the intercalating phenanthridine moiety. This cytotoxicity is possibly via the futile cycling of the nitro group with the concomitant production of reactive oxygen species.

The present results with P3 (P1) do not agree with our previous studies in which significant cytotoxicity was observed at a drug concentration of 0.5 mmol $dm^{-3}$. This cytotoxicity was equal towards both hypoxic and aerobic cells and, based on these results, the aerobic cytotoxicity of the NLP compounds was attributed to the intercalation of the targeting moiety into DNA (Cowan *et al.*, 1991). The lack of cytotoxicity observed with P3 (P1) in the present studies suggests a low level of contamination in the original preparation of P1, with the likely candidate being phenanthridine, the starting material in the synthesis. Controls performed with the original and subsequent batches of the NLPs, in which essentially identical cytotoxicity and sensitisation results were observed between the batches (data not shown), suggests that this contamination was not a confounding factor in results reported previously for the NLP compounds.

All of the NLP compounds are potent radiosensitisers of hypoxic cells *in vitro* based on external drug concentrations. Over the range of 2–4 carbons in the linking chain, almost the full oxygen effect can be achieved at an extracellular drug concentration of 0.5 mmol $dm^{-3}$ (Table III; Cowan *et al.*, 1991). As the chain length is increased further, a slight reduction in sensitising efficiency is observed (Figure 4). Therefore, rather than allowing the compounds to more efficiently 'scan' the DNA for target radicals as proposed, the longer chains appear to act by simply holding the nitroimidazole away from its target, resulting in a lower level of sensitisation. All the NLP drugs with linking chains of fewer than five carbons produce a small but reproducible radioprotection of aerobic cells (data not shown). One plausible explanation for this is that the nitroimidazole protruding from the DNA helix acts as a trap for the reactive water species produced by the radiation preventing them from reaching the DNA and causing damage. Another possible protection mechanism is that the NLP compounds may slow progression through the cell cycle allowing for an increase in the time available for the cells to repair radiation-induced damage, as has been previously postulated for the DNA-binding radioprotector, Hoechst 33342 (Smith & Anderson, 1984). Lowering the external drug concentration used in these experiments resulted in reductions in the level of both hypoxic cell sensitisation and protection of aerobic cells (data not shown). Depending on the drug concentration used, the NLP compounds are 10–100 times more effective as hypoxic cell sensitisers than the untargeted 2-nitroimidazole Miso based on external drug concentrations. None of the P series altered the radiation response of hypoxic or aerobic cells (Table IV), demonstrating that the NLPs act by an electron affinic radiosensitisation mechanism.

As shown previously for 2-NLP-3 (NLP-1) (Cowan *et al.*, 1991), the SER *vs* drug concentration curve is steeper for the NLP compounds than it is for Miso. This is in contrast to what has been seen previously for a 2-nitroimidazole linked to a quaternary base (a morpholine group) by a four-hydrocarbon chain where a shallower SER *vs* drug concentration curve was seen compared to Miso (Adams *et al.*, 1980*a*). This difference likely reflects the effect of targeting the 2-nitroimidazole to DNA via the intercalating phenanthridine of the NLP compounds.

Previous work by Adams *et al.* (1980*b*) has studied the dependency of the sensitising efficiency of 2-nitroimidazoles on the length of the hydrocarbon chain, linking them to positively charged, non-intercalating morpholine groups. Radiosensitising efficiency increased as the chain was lengthened, reaching a maximum at 4–5 carbons, and then decreased for linking chains up to 11 carbons in length. The chronic cytotoxicity of the morpholine group increased monotonically with chain length. The present results for the NLP compounds differ from these results in that radiosensitising efficiency was constant over 2–4 carbons and then decreased as the chain was lengthened to five and six carbons, while chronic cytotoxicity was at a minimum at four carbons. Clearly, linking a DNA targeting group to a 2-nitroimidazole via hydrocarbon chains of varying lengths produces results different from those seen with the attachment of a non-intercalating group. The causes of these differences remains to be determined.

Evidence of the ability of the NLP compounds to pass through several cell layers has been obtained *in vitro* using the multicellular spheroid tumour model. Studies with intact *vs* disassociated spheroids demonstrated significant cytotoxicity in both systems, with the spheroid cytotoxicity being intermediate between that seen for the single cells under aerobic compared with hypoxic exposure conditions (Figure 4). Assuming a diffusion distance for oxygen of 150 $\mu$m (Thomlinson & Gray, 1955; Tannock, 1972) and a V79 cell size of 10–15 $\mu$m, the compound would have to diffuse through 10–15 cell diameters to reach the hypoxic cells. Since the cytotoxicity observed in the intact spheroid case is greater than can be explained through aerobic killing alone, penetration of 2-NLP-3 (NLP-1) through multiple cell layers does not appear to be a major limitation in this class of compounds. Further preliminary evidence to support this result comes from a fluorescence-activated cell sorting technique in which spheroids are stained with the DNA dye Hoescht 33342, exposed to the drug, disassociated and sorted as a function of fluorescence intensity into four fractions. The cells are then plated and assayed for clonogenic survival. A similar pattern of cytotoxicity was seen in this system with the dimmest and therefore presumably most hypoxic fraction demonstrating significant cytotoxicity, again suggesting that 2-NLP-3 (NLP-1) could penetrate to this region (D.S.M. Cowan, unpublished results).

The *in vivo* toxicity of 2-NLP-3 (NLP-1) is approximately 10-fold greater than that seen for Miso. The acute toxic effects seen after administration of either 2-NLP-3 (NLP-1) or Miso were similar and appeared to include neurological involvement. Animals surving 1–2 h post administration suffered no deleterious complications, as has been seen previously for Miso (Rauth *et al.*, 1980).

Regrowth delay assays demonstrated significant activity in the SCCVII/To tumour (Figure 6), indicating that the NLP compounds are capable of penetrating through the tumour to reach the hypoxic cells and overcoming one of the limitations suggested for the use of other DNA targeted compounds *in vivo* (Roberts *et al.*, 1990; Denny *et al.*, 1992). Sensitisation of hypoxic cells equal to that seen with Miso was achieved at 2-NLP-3 (NLP-1) concentrations approximately 20-fold lower than Miso on a molar basis. Lowering the concentration further, to about 1/40th that of Miso, still produced sensitisation of hypoxic cells albeit to a more modest extent. However, since 2-NLP-3 (NLP-1) is approximately 10-fold more toxic than Miso, it may be no better than Miso in sensitising tumours when therapeutic ratios are compared. A complete dose–response comparison between Miso and 2-NLP-3 (NLP-1) is an important extension of this work and those studies are currently under way. Post-irradiation injection of 2-NLP-3 (NLP-1) produced no significant delay in tumour regrowth over that seen with radiation alone (data not shown). In addition, no anti-tumour effect was observed when the compound was administered in the absence of radiation (data not shown). These results suggest that at least the majority of the effect observed *in vivo* is due to radiosensitisation by the DNA-targeted compound.

The present studies suggest that targeting electron affinic compounds to DNA is an effective way of increasing their hypoxic cell cytotoxicity and radiosensitising potency *in vitro* and indicates the potential utility of this approach *in vivo*. Whether it is possible to further reduce the normal tissue cytotoxicity of such compounds, increase their sensitising potency *in vivo* or both enough to make them potential candidates for clinical evaluation remains unclear. Possible modifications to the compounds include alterations at both the electron affinic end as well as the targeting end and both these possibilities are being investigated.

The authors would like to thank Dr R.P. Hill and Dr I.F. Tannock for helpful discussions and Mr R.M. Kuba for technical advice. Work supported by The Medical Research Council of Canada and The National Cancer Institute of Canada.

### References

ADAMS, G.E., AHMED, I., CLARKE, E.D., O'NEILL, P., PARRICK, J., STRATFORD, I.J., WALLACE, R.G., WARDMAN, P. & WATTS, M.E. (1980*a*). Structure–activity relationships in the development of hypoxic cell radiosensitizers. *Int. J. Radiat. Oncol. Biol. Phys.*, **38**, 613–626.

ADAMS, G.E., AHMED, I., FIELDEN, E.M., O'NEILL, P. & STRATFORD, I.J. (1980*b*). The development of some nitroimidazoles as hypoxic cell sensitizers. *Cancer Clin. Trials*, **3**, 37–42.

BROWN, J.M. (1984). Clinical trials of radiosensitizers: what should we expect? *Int. J. Radiat. Oncol. Biol. Phys.*, **10**, 425–429.

BROWN, J.M. & WORKMAN, P. (1980). Partition coefficient as a guide to the development of radiosensitizers which are less toxic than misonidazole. *Radiat. Res.*, **82**, 171–190.

BUNTING, J.W. (1979). Heterocyclic pseudobases. *Adv. Heterocycl. Chem.*, **25**, 1–82.

COWAN, D.S.M., PANICUCCI, R., MCCLELLAND, R.A. & RAUTH, A.M. (1991). Targeting radiosensitizers to DNA: nitroimidazole-linked phenanthridines. *Radiat. Res.*, **127**, 81–89.

COWAN, D.S.M., KANAGASABAPATHY, V.M., MCCLELLAND, R.A. & RAUTH, A.M. (1992). Mechanistic studies of enhanced *in vitro* radiosensitization and hypoxic cell cytotoxicity by targeting radiosensitizers to DNA via intercalation. *Int. J. Radiat. Oncol. Biol. Phys.*, **22**, 541–544.

DENNY, W.A., ROBERTS, P.B., ANDERSON, R.F., BROWN, J.M. & WILSON, W.R. (1992). NLA-1: a 2-nitroimidazole radiosensitizer targeted to DNA by intercalation. *Int. J. Radiat. Oncol. Biol. Phys.*, **22**, 553–556.

MORGAN, A.P., LEE, J.S., PULLEYBLANK, D.E., MURRAY, N.L. & EVANS, D.H. (1979). Review: ethidium fluorescence assays. Part 1. Physicochemical studies. *Nucleic Acids Res.*, **7**, 547–569.

PANICUCCI, R., HEAL, R., LADEROUTE, K.L., COWAN, D., MCCLELLAND, R.A. & RAUTH, A.M. (1989). NLP-1: a DNA intercalating hypoxic cell radiosensitizer and cytotoxin. *Int. J. Radiat. Oncol. Biol. Phys.*, **16**, 1039–1043.

RAUTH, A.M., PACIGA, J.E. & MOHINDRA, J.K. (1980). *In vivo* studies of the cytotoxicity of hypoxic cell radiosensitizers. In *Radiation Sensitizers*, Brady, L.W. (ed.) pp. 207–214. Masson Publishing: New York.

ROBERTS, P.B., DENNY, W.A., WAKELIN, L.P.G., ANDERSON, R.F. & WILSON, W.R. (1990). Radiosensitization of mammalian cells *in vitro* by nitroacridines. *Radiat. Res.*, **123**, 153–164.

SIEMANN, D.W., HILL, R.P. & BUSH, R.S. (1977). The importance of the pre-irradiation breathing times of oxygen and carbogen (5% $CO_2$ and 95% $O_2$) on the *in vivo* radiation response of a murine sarcoma. *Int. J. Radiat. Oncol. Biol. Phys.*, **2**, 903–911.

SKOV, K.A. (1989). Workshop report: DNA targeted hypoxic cell cytotoxins and radiosensitizers. *Int. J. Radiat. Biol.*, **56**, 387–393.

SMITH, P.J. & ANDERSON, C.O. (1984). Modification of the radiation sensitivity of human tumour cells by a bis-benzimidazole derivative. *Int. J. Radiat. Biol.*, **46**, 331–344.

TANNOCK, I.F. (1972). Oxygen diffusion and the distribution of cellular radiosensitivity in tumours. *Br. J. Radiol.*, **46**, 515–524.

THOMLINSON, R.H. & GRAY, L.H. (1955). The histological structure of some human lung cancers and the possible implications for radiotherapy. *Br. J. Cancer*, **9**, 539–549.

WEINBERG, M.J., LAPOINTE, T.A. & RAUTH, A.M. (1986). Growth delay in a murine squamous cell tumor after local radiation and concurrent infusional 5-fluorouracil treatment. *Int. J. Radiat. Oncol. Biol. Phys.*, **12**, 1449–1452.